# Comparison of the Performance Parameters of BioHPP^®^ and Biocetal^®^ Used in the Production of Prosthetic Restorations in Dentistry—Part II: Physicochemical and Microbiological Tests: An In Vitro Study

**DOI:** 10.3390/ma18030519

**Published:** 2025-01-23

**Authors:** Robert Kowalski, Wojciech Frąckiewicz, Magdalena Kwiatkowska, Marcin Adamiak, Agata Pruss, Ewa Sobolewska

**Affiliations:** 1 Department of Dental Prosthetics, Faculty of Medicine and Dentistry, Pomeranian Medical University in Szczecin, Av. Powstańców Wlkp. 72, 70-111 Szczecin, Poland; 2 Ra-Dent Stomatologia Protetyka, Bolesława Krzywoustego Street 19/5, 70-252 Szczecin, Poland; 3 Faculty of Mechanical Engineering and Mechatronics, West Pomeranian University of Technology in Szczecin, Av. Piastów 19, 70-310 Szczecin, Poland; 4 Materials Testing Laboratory—RMT L1, Faculty of Mechanical Engineering, Silesian University of Technology, Konarskiego Street 18a, 44-100 Gliwice, Poland; 5 Department of Laboratory Medicine, Faculty of Medicine and Dentistry, Pomeranian Medical University in Szczecin, Av. Powstańców Wlkp. 72, 70-111 Szczecin, Poland; agata.pruss@pum.edu.pl

**Keywords:** dentistry, Biocetal, BioHPP, density, water absorption, contact angle, surface roughness, SEM, bacteria

## Abstract

The natural aging process of the human organism leads to both physiological and pathological changes, including tooth loss. This requires dental prosthetic interventions aimed at restoring patients’ quality of life. The use of such prostheses necessitates selection of sufficiently strong, aesthetic and biocompatible materials, which also offer ease of shaping. The market for materials used in prosthetic applications offers a wide array of options; however, selection of the most suitable material for specific clinical scenarios can be challenging for dental professionals. This paper continues the comprehensive investigation of the physiochemical and mechanical/functional properties of two commonly used prosthetic—Biocetal and BioHPP—offering a comparative analysis of their characteristics to provide valuable insights for dentists and prosthodontists. The study focuses on in vitro analyses of physiochemical parameters, including density, water sorption, contact angle, and surface roughness. The structure of the materials was examined via scanning electron microscopy. Additionally, microbiological studies were performed using strains of *Staphylococcus aureus*, *Enterococcus faecalis*, *Escherichia coli*, *Pseudomonas aeruginosa* and *Candida albicans*. Statistical analysis was performed using Shapiro–Wilk test, Q-Q plot analysis, Grubbs test, and Student’s T-test (*p* < 0.05). The findings indicate that BioHPP demonstrates superior physiochemical and microbiological properties. However, Biocetal exhibit better surface characteristics. Despite its high performance, BioHPP presents certain drawbacks, which may influence dentists’ material choice in specific clinical cases, particularly for certain prosthetic restorations.

## 1. Introduction

The material used to manufacture dentures should be characterized not only by high mechanical parameters but also by appropriate physicochemical and surface features to ensure long-term durability of the restoration. One study [1] showed that acetal material has lower water solubility and sorption compared to poly(methyl methacrylate), PMMA. This is particularly important when acetal is used to construct dentures used in the oral cavity for a long time, because the absorption of fluids can cause changes on the surface of these restorations. The color of food, its pH, and consequently the pH of saliva can affect the discoloration of the material used for the construction [2]. Using artificial saliva, authors confirmed that acidic pH causes acetal material to lighten, while alkaline pH causes a darker color compared to the original color.

Another important feature is the biocompatibility of materials used in dentistry. The susceptibility of dentures to the accumulation of microorganisms on their surface is also a disadvantage that patients and doctors pay special attention to. Due to the increased deposition of bacteria and fungi on removable dentures, the oral mucosa is exposed to the occurrence of prosthetic stomatopathy [3]. It has been proven that in the oral cavity, microorganisms *Candida albicans*, *Pseudomonas aeruginosa*, *Staphylococcus aureus*, *Enterococcus hirae* and *Escherichia coli* bacteria colonize and settle on acetal material to the least extent compared to acrylic resin and chromium–cobalt alloy [4,5]. A study conducted on Wistar rats on the biocompatibility of acetal material [6,7,8,9] showed that in the histopathological analysis, slight inflammatory reactions were observed in places in contact with the plates. However, further, more detailed clinical and laboratory studies are still required to confirm the inertness of acetal material.

Biocetal is a thermoplastic material intended for injection or compression molding, consisting of a polyoxymethylene homopolymer (POM). The main purpose of this material is the production of metal-free skeletal dentures, as well as retention elements. Polymers called polyacetals are formed as a result of polymerization of aldehydes. The most popular of them is polyoxymethylene (POM), which can be synthesized in two forms. The first one—copolymer (POM_C)—is formed from trioxane, while the second one—homopolymer (POM_H), like acetal resin used in dental prostheses—is synthesized via anionic polymerization of formaldehyde, also known as formaldehyde. Homopolymer macromolecules consist of alternating methyl groups connected by an oxygen atom O [10,11,12,13,14,15]. High degree of crystallinity makes processing of POM difficult. Moreover, due to different densities and thermal expansion coefficients between amorphous and crystalline phases, significant shrinkage and formation of voids occur [16].

BioHPP is a semi-crystalline and high-quality composite based on polyetheretherketone (PEEK) containing 20% of special ceramic fillers (BioHPP; Bredent GmbH&Co. KG, Germany). The grain size of microfillers ranges from 0.3 to 0.5 µm, which ensures constant homogeneity of the material structure, which is essential for obtaining the highest quality chemical bond between the BioHPP substructure and the veneering material. The fine-grained filler makes the subsequent polishing process more effective, which prevents bacterial plaque from settling and ensures color stability while maintaining high gloss. Additionally, enriching the material with microceramics significantly improves such material properties as mechanical strength and resistance to abrasive wear. PEEK is a rigid material with excellent thermal stability [17]. The physicochemical parameters of this material indicate a solubility in water at room temperature of 0.5% by weight [18,19,20] and a density of 1.3–1.5 g/cm^3^ [21,22,23], which makes the material stable in the human body [24].

This material induces a slight immune response in a living organism, but studies [25,26] have excluded that PEEK has mutagenic and cytotoxic effects. PEEK material may also be an alternative to metals such as titanium [21,27].

Studies have shown [28] that the enhancement of the antibacterial properties of PEEK can be successfully achieved by coating the surface of the material with hydroxyapatite doped with silver ions. Cytocompatible PEEK/Nano-FHA biocomposite after surface treatment to increase the roughness of the material shows better bioactivity, osteointegration and contact between the bone and the implant made of PEEK compared to pure PEEK [29]. PEEK can also be used to make a substructure for fixed prosthetic restorations. In order to increase the aesthetics of such work, the façade surface of the substructure can be covered with a more aesthetic material such as dental composite [30]. PEEK is currently often the recommended material for patients allergic to titanium [31,32,33,34,35,36].

Biocetal dentures are suitable for complicated cases, where the production of a cast-metal removable partial denture made of steel or BioHPP is impossible. The high elasticity of Biocetal increases the possibilities of prosthetics of strongly rotated or tilted teeth, even in the complete absence of a guiding surface. Despite the higher strength parameters, the BioHPP material in such clinical situations requires significant grinding of the teeth, which is why the dentist must always decide which material should be used in a given clinical situation. Although PEEK has numerous advantages in the context of restorative dentistry, due to its high cost and limitations in certain physical properties, this material is still under investigation. PEEK may be a good alternative for various dental restorations, but more clinical experiments and a sufficient number of studies are still needed [34,37].

This paper continues the comprehensive investigation of the physiochemical and mechanical/functional properties of two commonly used prosthetics—Biocetal and BioHPP—offering a comparative analysis of their characteristics and providing valuable insights for dentists and prosthodontists. The study focuses on in vitro analyses of physiochemical parameters, including density, water sorption, contact angle, and surface roughness. The structure of materials was examined via scanning electron microscopy. Additionally, microbiological studies were performed using strains of *Staphylococcus aureus*, *Enterococcus faecalis*, *Escherichia coli*, *Pseudomonas aeruginosa* and *Candida albicans*. Statistical analysis was performed using Shapiro–Wilk test, Q-Q plot analysis, Grubbs test, and Student’s T-test (*p* < 0.05). Although both materials have been widely studied, mainly in reviews [21,27,38,39], the authors intended to determine the features of these materials in their own research in both parts of studies. The presentation of strengths and weaknesses is aimed to facilitate the appropriate material selection for a given clinical case.

## 2. Materials and Methods

In our studies, the following materials were subjected to characterization: Biocetal produced by ROKO Dental Systems, Częstochowa, Poland, and BioHPP produced by Bredent GmbH &Co. KG, Senden, Germany. The tested samples had the dog-bone shapes in accordance with the PN-ISO 37:2024 [40], type 3 standard [22], and they are presented in Supplementary Information (SI, Figure S1).

All samples were prepared in the injection molding process using a laboratory scale injection machine Boy 15 (Dr Boy, Neustadt-Fernthal, Germany) and injection mold designed to form standardized specimens. The processing parameters as well as preparation of materials for processing are described in Part 1 of this paper [Materials Part 1]. Before every physicochemical test, the samples of both materials were identically treated as required by specified research technique.

### 2.1. Density Test

Density (*ρ*) is one of the basic physical properties that characterize plastics. It is defined as the ratio of the mass of the plastic sample (*m*) to its volume (*V*) at a given temperature:

$$\rho =\frac{m}{V}[g/{cm}^{3}]$$

There are many methods of measuring density that can be used, depending on the shape of the tested object. In the case of objects and samples larger than granulate, the hydrostatic method is used [41]. The density of the tested materials was determined using a hydrostatic balance (RADWAG, Radom, Poland). It allows for measuring the density of samples with compact, irregular shapes in particular. The hydrostatic weighing method consists in weighing the tested solid in air (the upper weighing pan) and then in an immersion liquid of known density, e.g., H_2_O (the lower weighing pan).

The density of the sample was calculated automatically by the device. The basis for calculating the density is the following formula:

$$\rho_{o}=\frac{w_{a}\cdot \rho_{im}}{w_{a}-w_{im}}[g/{cm}^{3}]$$

where

*w_a_*—sample weight in air;

*w_im_*—sample weight in immersion liquid;

*ρ_im_*—density of immersion liquid.

The test used water at a temperature of 23 °C, having a density of 0.99756 g/cm^3^. The density of 5 samples of acetal plastic and 5 samples of BioHPP was measured.

### 2.2. Water Absorption Test

Water absorption is expressed in percentage and is defined as the ratio of the weight of water absorbed by the sample to the weight of the sample in a dry state [41]. It is defined by the following formula:

(1) $$W_{A}=\frac{w_{w}-w_{d}}{w_{d}}\cdot 100[\%]$$

where

*W_A_*—water absorption by weight;

*w_w_*—weight of the sample in a water-saturated state;

*w_d_*—dry sample weight.

For the water absorption test, 10 samples of acetal and 10 of BioHPP were used. All samples were first dried at 80 °C for an hour. After drying, the samples were weighed with an accuracy of 0.1 mg on an analytical balance by Radwag AS 160/C/2. Then, the water absorption was measured using two methods. The first one was the determination of water absorption in boiling water, in which 5 samples of both materials were boiled at 100 °C for 30 min and then placed in a vessel with water at room temperature for 15 min. After drying, the samples were weighed again.

The second method is the measurement of water absorption in cold distilled water, in which 5 samples of both materials were immersed in water for 24 h. After this time, the samples were dried and weighed.

### 2.3. Contact Angle

The wetting/contact angle (θ) is the angle formed by the tangent to the surface of the measuring droplet deposited on the surface of a solid, at the point of contact of three phases—solid (S), liquid (L) and gas (V). The smaller the wetting angle, the greater the wettability of the material with the given liquid.

**Figure 1:**
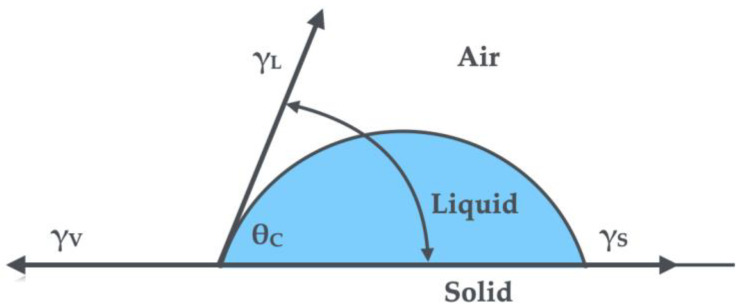
Scheme showing the three-phase boundary, where θ_C_—liquid contact angle, γ_L_—liquid surface free energy, γ_S_—solid surface free energy, γ_V_—air surface free energy.

Atoms located in the interphase region or at the boundary of phases (liquid, solid, gas) are subject to a different system of forces than atoms located deep in the phase. On the one hand, they are attracted by the atoms of their own phase, and on the other hand by the atoms of the neighboring phase. Surface tension (σ) and surface free energy (γ) are parameters describing the equilibrium state of atoms at the phase boundary (Figure 1).

Wettability is the process of covering a surface with a liquid layer in a given atmosphere. Such a layer is formed as a result of the interaction of surfaces that prefer contact with the liquid phase. If a given surface is well wetted by a given liquid, we say that it is hydrophilic, while surfaces that are not wetted by given liquids are called hydrophobic. The higher the surface free energy of a material, the better the liquid wets the surface.

In order to determine the wettability of the surfaces of the tested samples, wetting angle (θ) measurements were performed using the sessile drop method (static), i.e., 5 s after the drop was applied to the sample, and the dynamic method, which consisted in measuring the advancing (growing) wetting angle during drop dispensing, and then the retracting (decreasing) angle during piston retraction and drop suction back into the syringe. Additionally, the angle hysteresis was determined in this way, i.e., the difference between the increasing and decreasing wetting angle.

To measure the angle using the static method, a measuring system consisting of a Surftens Universal goniometer (OEG, Hessisch Oldendorf, Germany) and a computer with Surftens 4.5 software was used.

Measuring drops of 1.5 µL of liquid were deposited on the surface of the tested samples, while maintaining the minimum (same) height of the needle above the surface each time. Automatic measurements of the contact angle in a series of 10 measurements for each sample were taken after 5 s from the moment of depositing the liquid drop on the surface. The measurements were carried out at a standard temperature of 289 K (25 °C). The Owens–Wendt method was used to calculate the Surface Free Energy (SFE) and its components, i.e., polar and dispersive, which consists in measuring the contact angle using two liquids, one polar and the other nonpolar. The method assumes that the Surface Free Energy (*γ_S_*) of a solid is the sum of the dispersive component (*γ_S_^d^*) and the poured component (*γ_S_^p^*) according to the following equations:

$$\gamma_{S}=\gamma_{S}^{d}+\gamma_{S}^{p}$$

$$\gamma_{L}\left(1+cos\theta \right)=2\sqrt{\gamma_{S}^{d}\cdot \gamma_{L}^{d}}+2\sqrt{\gamma_{S}^{p}\cdot \gamma_{L}^{P}}$$

where

$\gamma_{\frac{S}{L}}^{d}$—solid/liquid dispersion component;

$\gamma_{\frac{S}{L}}^{p}$—polar component of solid/liquid;

*θ*—wetting angle.

In the method, the liquids should be selected so that one has a high γ_L_^d^ value and a low γ_L_^p^ value. Therefore, distilled water (polar liquid) and diiodomethane (dispersive liquid) were used in the tests as measuring liquids (Table 1).

In this way, the contact angle was measured using the static method 10 times on 5 Biocetal samples and 10 times on 5 BioHPP samples, after prior degreasing of the samples with isopropyl alcohol.

In order to determine the wettability of the surfaces of the tested samples, wetting angle (θ) measurements were also carried out using the dynamic method. For this purpose, a DSA 100 goniometer (Krüss, Hamburg, Germany) was used (SI, Figure S2).

**Figure 2:**
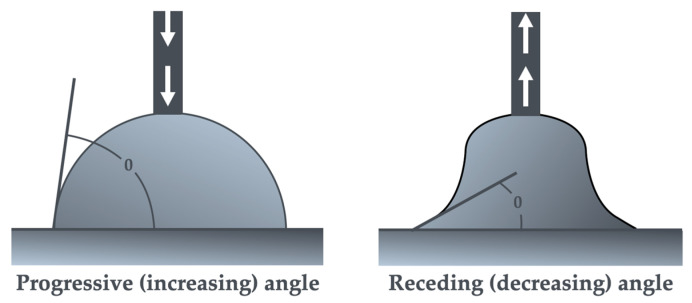
Diagram showing the progressive and receding angle.

A drop of ultrapure water with a volume of 1.5 µL was deposited on the surface of the tested samples, and then its volume was increased to 10 µL. During this time, the camera recorded the image and the advancing wetting angle was measured. After reaching 10 µL of volume, the syringe started to retract the piston and suck the drop back into 2 µL of liquid, measuring the receding wetting angle (Figure 2). Then, the difference between the progressive and receding angles (wetting angle hysteresis) was calculated.

In this way, the wettability angle was measured 6 times on 3 Biocetal samples and 6 times on 3 BioHPP samples, after prior degreasing of the samples with isopropyl alcohol.

### 2.4. Surface Roughness

Roughness is a feature of the surface of a solid body, indicating recognizable optical or mechanically perceptible surface irregularities, resulting not from its shape but from the nature of processing and the tool used. Surface roughness assessment, unlike waviness assessment, is performed on short elementary sections of length from 0.08 mm to 8 mm. Roughness is most often expressed using two parameters: *R_a_* and *R_z_*.

*R_a_*—it is the arithmetic mean of the absolute values of the ordinates of the elementary section. This parameter reacts poorly to local changes and does not provide a clear picture of the surface condition.

*R_z_*—it is the maximum height of the roughness profile. It is the sum of the arithmetic mean height of the five highest peaks above the mean line and the mean depth of the five lowest depressions below the mean line.

*R_t_*—when describing roughness, the total height of the profile is also given, which is the sum of the height of the highest peak and the depth of the lowest depression within the measuring section. This parameter is the only one that is calculated for the entire measuring section, not for the elementary section.

Together with the roughness measurement, the surface topography of the samples was also observed using a Leica DVM6 digital microscope (Leica Microsystems, Wetzlar, Germany). Additionally, a scan of the sample surface was performed to obtain a surface topography map. Roughness measurements were made on 5 Biocetal samples and 5 BioHPP samples. They were performed using a Surtronic 25 Profilometer (Taylor-Hobson, Leicester, Great Britain, UK) for a measuring section of 2.5 mm. The analysis of the roughness profiles was performed using the TaylorProfile program.

### 2.5. Structure Examination Using Scanning Electron Microscopy

The structure of the investigated materials was analyzed using scanning electron microscope, SEM (JEOL JSM 6100 SEM ULTRA 55, Tokyo, Japan). To prepare the samples, it was necessary to perform a brittle fracture. An attempt to break or cut the samples without preliminary preparation results in stretching or destruction of the materials, which prevents accurate observation. Therefore, the samples were cooled in liquid nitrogen, and then a brittle fracture was performed, which occurs without macroscopic plastic deformations. It is caused by a load that exceeds the cohesion of the material.

Scanning a sample generates an electric charge on its surface; therefore, for correct detection of the SEM image, the samples must have appropriate conductivity. Otherwise, not only is it not be possible to obtain the correct measurement, but the sample may also be damaged. Standardly, samples are covered with a thin layer of gold or carbon, among others. The simplest coating method is resistive sputtering, which involves the deposition of thin layers, the source of which is a heated, refractory material. This method was applied in our own study, and the vacuum evaporator (JEOL JEE-4X, Tokyo, Japan) was used to cover the samples with a thin and uniform layer of gold (SI, Figure S3).

### 2.6. Microbiological Examination

The aim of the study was to assess the adhesion of bacteria and fungi of the *Candida albicans* genus to the surfaces of tested materials used in the production of dental prostheses.

Studies were conducted on 5 species of test microorganisms: *Staphylococcus aureus*, *Enterococcus faecalis*, *E. coli*, *Pseudomonas aeruginosa* and *Candida albicans*. The strains were inoculated on appropriate media, and after 18 h of cultivation, the strains were suspended in a physiological saline solution. One sterilized sample of the prosthetic material was placed in each suspension with microorganisms. The control sample consisted of samples suspended in physiological saline. The plates with the bacterial and yeast suspension and the control plates were incubated for 60 min at 37 °C, shaking the suspension every 15 min. After incubation, the samples were washed three times in a NaCl solution. The removed and dried plates were transferred to bacteriological media for 1 min. The cultures were incubated for 24 h at 37 °C, after which the colonies that grew at the imprint site were counted. The test was performed in 3 replicates, on 6 samples of each material, including one control sample. The adhesion and the number of colonies on the individual materials were assessed.

### 2.7. Statistical Analysis

The obtained results are presented in tables and graphs for each experiment and material, whilst the results of statistical analyses are collected in tables included in Supplementary Information. For each variable, the number of samples used, the minimum and maximum values from the measurements, the median, the first and third quartile, the mean, the standard deviation and the standard error are presented. The Shapiro–Wilk test was used to assess the normality of the distribution of the studied variables. In the case of deviations from the normal distribution, the variable was assessed for the occurrence of outliers using the Q-Q plot analysis and the Grubbs test. In the case of no significant deviation from the normal distribution of variables in both groups, Student’s T test was used for comparisons between groups. The assumption of homogeneity of variance was verified using the Levene test; if it was not met, Student’s T test with Welch’s correction was used. The analysis was performed in the R language in the RStudio environment using the tidyverse package. The results of statistical analyses were considered statistically significant at a *p* value of <0.05.

## 3. Results

### 3.1. Density Measurement Results

A significantly higher density of BioHPP material was observed compared to Biocetal. The average density of BioHPP was 1.49 ± 0.01 g/cm^3^, while the average density of Biocetal was 1.41 ± 0.01 g/cm^3^ (Table 2; SI, Table S1). The higher density of BioHPP may also result from the presence of ceramic filler.

### 3.2. Water Absorption Results

Both in cold and hot water, Biocetal was characterized by significantly higher water absorption values than BioHPP. The average percentage mass increase in boiling water was 1.27 ± 0.05% and 0.41 ± 0.25% for Biocetal and BioHPP, respectively (Table 2, SI Table S2), while in cold water the percentage mass increase was 0.63 ± 0.05% and 0.11 ± 0.02% for Biocetal and BioHPP, respectively (Table 2, SI Table S3).

### 3.3. Contact Angle Results

**Figure 3:**
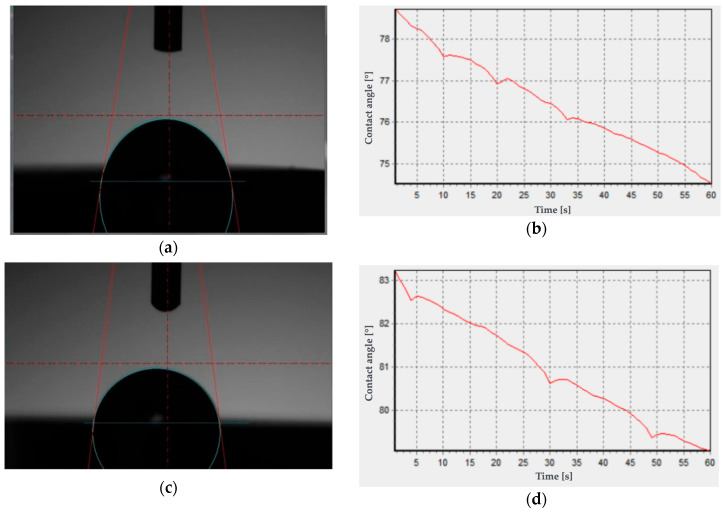
Example results of contact angle measurements with distilled water: (**a**) Biocetal—measuring drop, (**b**) Biocetal—graph of contact angle changes as a function of time, (**c**) BioHPP—measuring drop, (**d**) BioHPP—graph of contact angle changes as a function of time.

The determined average values of the contact angle with distilled water are less than 90° (Table 3, Figure 3). Based on the calculations of surface free energy, it was shown that the average values for both test groups of Biocetal and BioHPP are in the range of 40 to 42 mJ/m^2^ and show increased affinity to the SEP dispersion groups (Table 3).

The static contact angle for Biocetal and BioHPP was performed using two liquids—water and diiodomethane. The obtained values were 85° and 84° for water for Biocetal and BioHPP and 40° and 37° for diiodomethane for Biocetal and BioHPP, respectively.

Additionally, to confirm the correctness of the measurement, the wetting angle was measured for 60 s using distilled water (Figure 3b,d). No significant change in the wetting angle value was noted for 60 s, which proves the correctness of the measurement and the stability of the droplet.

Table 4 presents the results of the dynamic wetting angle test, in which the advancing (increasing) and receding (decreasing) wetting angle were determined and the angle hysteresis was determined.

A difference was observed between the static (Table 3) and dynamic (Table 4) wetting angles of the materials. This difference may be influenced by the surface free energy (SFE) of the materials and the roughness of the materials.

### 3.4. Roughness Results

**Figure 4:**
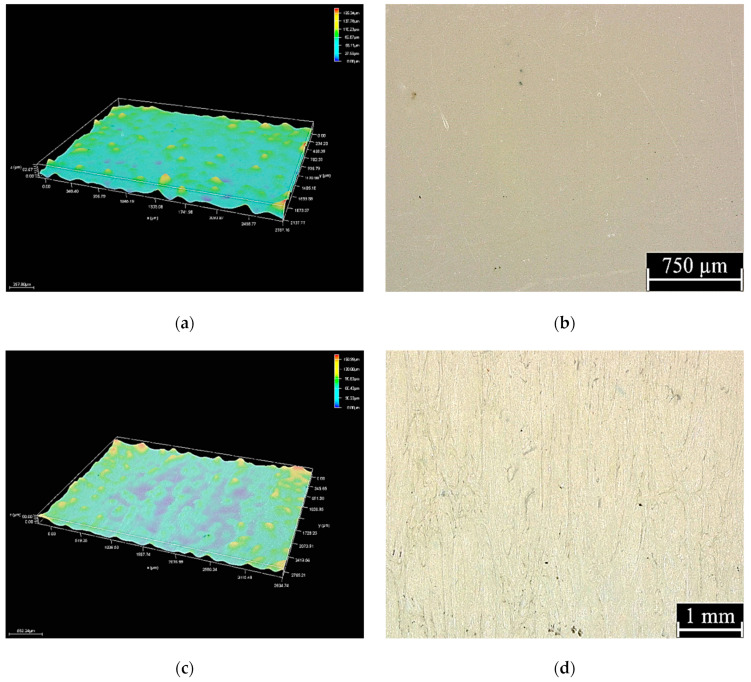
Surface of Biocetal ×100 (**a**,**b**) and BioHPP ×75 (**c**,**d**) samples.

Based on the observations and microscopic analyses performed using a Leica DVM6 digital microscope (Leica Microsystems, Germany), it was shown that the surface of the Biocetal sample was uniform and free from defects. In the case of BioHPP material samples, the presence of greater inhomogeneity of the tested surface was revealed, which results from the structure of the material: it is a composite which contains particles with a lamellar geometry and micrometric dimensions (Figure 4). The roughness measurements are shown in Table 5.

No significant differences were observed between Biocetal and BioHPP in terms of the arithmetic mean deviation from the mean roughness line (*R_a_*) (SI, Table S4) as well as in terms of the highest roughness height according to the measured 10 highest profiles *(R_z_)* (SI, Table S5). Moreover, Biocetal and BioHPP did not differ significantly in the total roughness profile height (*R_t_*) (SI, Table S6).

**Figure 5:**
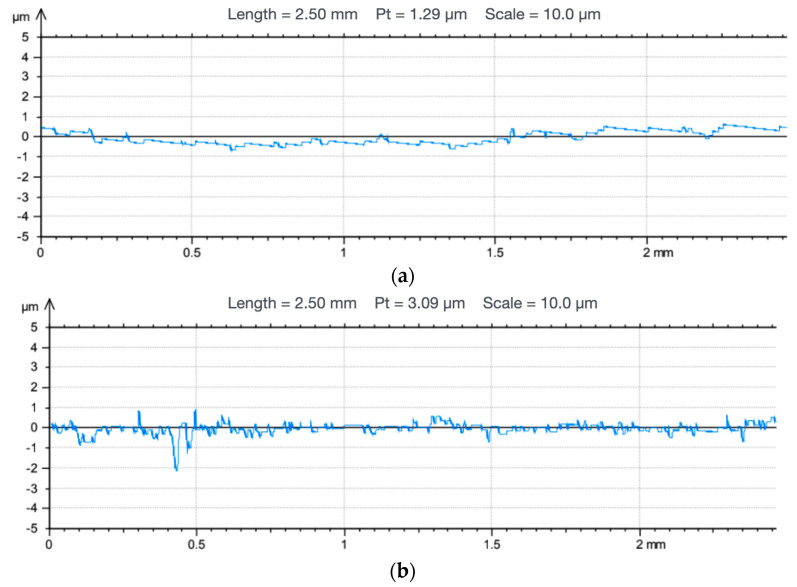
Surface roughness profiles of (**a**) Biocetal and (**b**) BioHPP.

Based on the analyses and measurements, it was shown that BioHPP samples are characterized by higher surface roughness in comparison to Biocetal samples. Roughness profile measurements (Figure 5) indicate surface development (presence of unevenness), which corresponds to images recorded using microscopic techniques.

### 3.5. Results of Sample Structure Examination Using SEM

**Figure 6:**
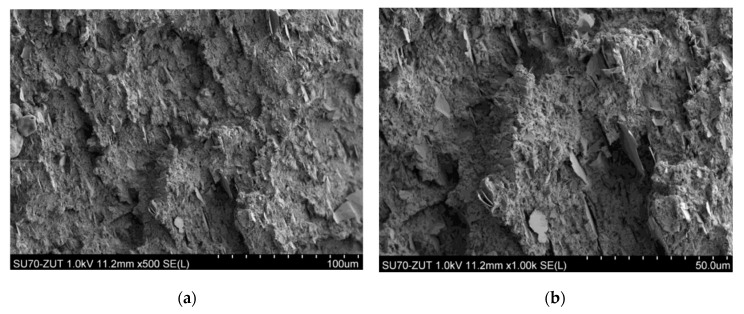

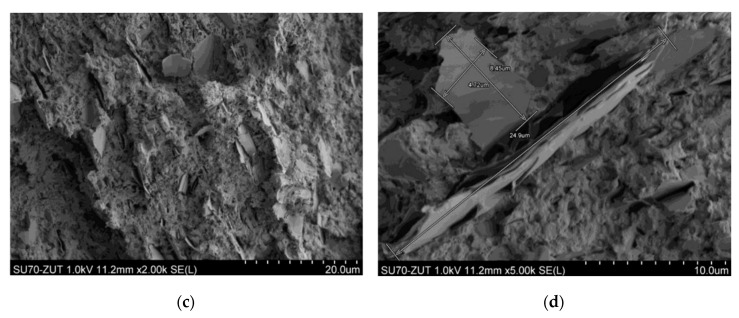
BioHPP sample. Clusters of ceramic microfillers are visible. (**a**) ×500 zoom; (**b**) ×1000 zoom; (**c**) ×2000 zoom; (**d**) ×5000 zoom.

**Figure 7:**
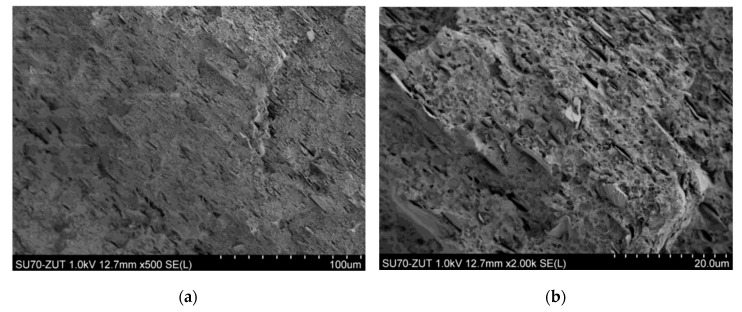
SEM image of the opposite part of the BioHPP brittle fracture. The cavities from the broken clusters of ceramic microfiller are visible. (**a**) ×500 magnification; (**b**) ×2000 magnification.

**Figure 8:**
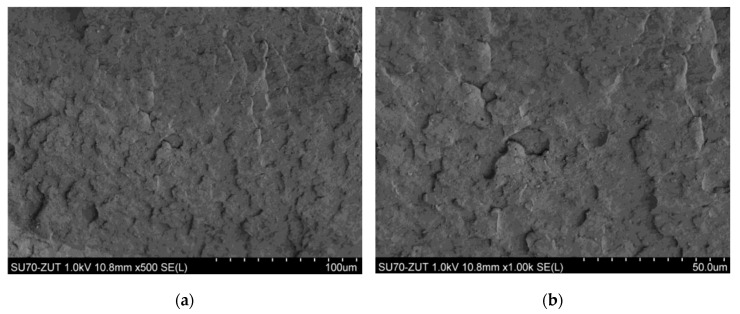

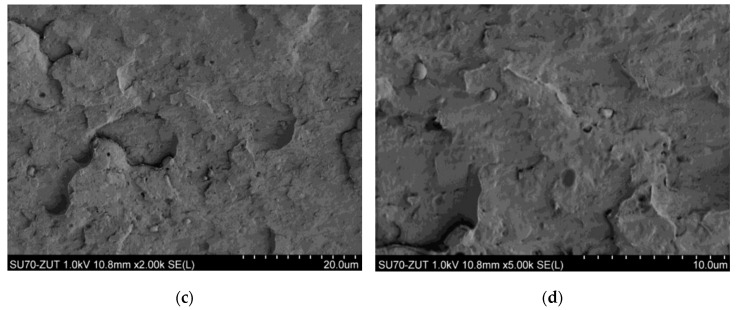
SEM image of the Biocetal sample. A more homogeneous surface of the material is visible compared to BioHPP. (**a**) ×500 approximation; (**b**) ×1000 approximation; (**c**) ×2000 approximation; (**d**) ×5000 approximation.

When observing the BioHPP sample using SEM microscope, one can see clusters of ceramic microfiller (Figure 6 and Figure 7). They appear in the form of “clusters”, i.e., glued plates with a size of several to a dozen or so micrometers. In the case of Biocetal, a more homogeneous structure is visible (Figure 8).

The difference between the materials studied is that Biocetal is a homopolymer and BioHPP is a composite, which is why they differ in fracture structure. The filler particles have a lamellar structure, differ in surface size and are distributed quite uniformly, although larger clusters are also observed. On the other hand, the topography of the Biocetal cross-section results from the nature of the fracture. In the case of this material, there are no particles, and the observed spherical inclusions may be particles of catalysts or other refining additives.

### 3.6. Microbiological Test Results

The results of microbiological tests are presented in Table 6 and Table S7.

## 4. Discussion

Determining the differences in the physicochemical and mechanical properties of materials used in the production of dental prostheses is a very important element of the analysis of the usability of these materials. Selecting the appropriate material for the production of prosthetic restorations in a given clinical case, which is both aesthetic and acceptable to the patient, and resistant to the conditions prevailing in the oral cavity, requires the doctor to have knowledge of the physicochemical and mechanical properties of a given material in comparison to others.

Our own study aimed to compare the properties of Biocetal and BioHPP. Both materials in the form of granules were prepared and processed in the same way using an industrial injection molding machine. Each study of physicochemical, surface and microbiological properties, as well as mechanical properties from the first part of the study was preceded by identical preparation of samples made of both materials. Therefore, the influence of sample processing on the differences in the obtained results between the materials was rejected.

The average density of BioHPP in our own studies was 1.49 ± 0.01 g/cm^3^ which, according to statistical analysis, is a value significantly higher than the density of Biocetal, which was 1.41 ± 0.01 g/cm^3^. The density of Biocetal given by the manufacturer in the safety data sheet is 1.4 ± 1.45 g/cm^3^ [42], which corresponds to the density measured in our own study. In comparison with other materials used in dentistry, such as chromium-cobalt alloys or silver–palladium (10–12 g/cm^3^), the obtained density results of the tested materials are still significantly lower. The low density of both tested materials in comparison with metals makes the prosthesis made of them light and user-friendly. Comparing the density of the materials with each other, the higher density of BioHPP affects its lower translucency, water absorption, as well as its higher stiffness and resistance to color change.

The water absorption of prosthetic materials is one of the key factors that should be taken into account when designing a restoration and selecting prosthetic materials, because water that is absorbed by the material can affect its structure and lead to a decrease in mechanical strength, deterioration of its quality and durability, and changes in physical and chemical properties. Due to the fact that prosthetic materials are used in the moist environment of the oral cavity and are exposed to contact with saliva, their absorption is very important from the point of view of oral hygiene. High absorption of materials can promote the growth of bacteria on their surface and the formation of pathological changes in the entire oral cavity.

BioHPP showed lower water absorption in both boiling and cold water. The average percentage mass gain in boiling water was 1.27 ± 0.05% and 0.41 ± 0.25% for Biocetal and BioHPP, respectively, while in cold water the percentage mass gain was 0.63 ± 0.06% and 0.11 ± 0.02% for Biocetal and BioHPP, respectively. The water absorption given by the manufacturer of BioHPP [43] is <6.5 μg/mm^3^, and the water solubility is <0.3 μg/mm^3^.

These values cannot be compared precisely with those obtained in our own study, because they differ in methodology. Manufacturer BioHPP (Bredent, Senden, Germany) provides values referring to weight gain or loss per mm^3^, while in our own study the percentage weight gain measured on an analytical balance was specified.

In the study by Arikan et al. [1], the sorption and solubility in water of white and pink acetal resin were studied and compared with PMMA. For this purpose, pre-dried samples in the form of disks with a diameter of 50 ± 0.1 mm and a thickness of about 0.5 mm (with a weight of *m*_1_ determined in the study) were immersed for 7 days in distilled water at a temperature of 37 ± 2 °C. After removing and drying the samples with paper and an air stream, they were weighed (m^2^), and in this way an average water sorption of 12.99 μg/mm^3^ for the pink resin, 17.03 μg/mm^3^ for the white resin and 17.99 μg/mm^3^ for PMMA was obtained. Then, the same samples were used to measure the solubility in water. The samples were dried in special vessels called desiccators and weighed again (m^3^). Water solubility was 1.60 μg/mm^3^ for PMMA, 0.11 μg/mm^3^ for pink acetal resin and 0.13 μg/mm^3^ for white. Statistical analysis using the nonparametric Kruskal–Wallis test showed significantly lower sorption of pink acetal resin compared to white and PMMA. In the case of water solubility, PMMA showed significantly higher solubility compared to the other resins, and among them, pink acetal resin achieved higher solubility than white, but this difference was not statistically significant. The results of our own research cannot be precisely compared with the research of Arikan et al. because the results obtained in this study referred to the weight increase per mm^3^, while in our study the percentage weight increase measured on an analytical balance was determined.

Plastic materials used in dental prosthetics are exposed to the moist environment of the oral cavity. Liquids in contact with plastic can penetrate into the material and bind to the polymer by chemical or physicochemical and mechanical bonds, causing a decrease in the strength of intermolecular interactions and an increase in the mobility of macromolecules. This can result in a decrease in the modulus of elasticity, tensile strength and hardness of the material.

The contact angle depends on the surface tension of both the liquid and the solid surface. If the surface tension of the liquid is lesser than the surface tension of the solid surface, the liquid drop does not spread on the solid surface and the contact angle is greater than 90 degrees (the liquid does not wet the solid surface). However, if the surface tension of the liquid is greater than the surface tension of the solid surface, the liquid drop spreads on the solid surface and the contact angle is lesser than 90 degrees (the liquid wets the solid surface).

The contact angle is also a very important parameter that allows determining the wettability of materials used in dental prosthetics. The wetting angle test showed hydrophilic properties of both tested materials, whereby during the wetting angle test using the static method, Biocetal obtained a higher wetting angle result (85.3°) compared to BioHPP (84.1°), while in the dynamic method the value of the progressive wetting angle of BioHPP was higher (90.6°) than Biocetal (84.7°). The calculated Surface Free Energy of BioHPP was 42 ± 0.7 mJ/m^2^, while for Biocetal it was 40 ± 0.3 mJ/m^2^. The higher surface energy of BioHPP affects its low wetting angle in the static test.

The difference in the values of static and dynamic angles is justified by the difference in physical factors influencing these measurements. The static angle measurement was performed 5 s after the drop was applied to the sample. The boundary of three phases, liquid, solid (sample) and gas, was stabilized at this time, and the obtained wetting angle result was dependent on the surface free energy (SEP) of the sample. In the case of the dynamic angle, the progressive wetting angle was measured as a result of a constant increase in the volume of a water droplet to 10 µL, while the drop front was constantly moving over the sample, encountering a previously unwetted, rough surface.

In the study of the dynamic wetting angle, the phenomenon of wetting angle hysteresis was observed. It consists in the fact that the advancing wetting angle is different from the receding angle. This is related to the roughness of the material surface and depends on the point at which the liquid surface front stops. For surfaces with very low roughness, the hysteresis is very small, which means that the advancing angle has a value close to the receding angle. In our own study, the angle hysteresis for Biocetal was 33.2°, while for BioHPP it was 44.1°, which may result from the fact that BioHPP is a rougher material. However, it must be taken into account that the measurements of the wetting angles of both materials were performed on materials without mechanical processing, i.e., without smoothing and polishing the surface. The samples were only degreased using isopropyl alcohol.

Due to the presence of microorganisms in the oral cavity, it is important that the prosthetic material is well polished in order to avoid the penetration of bacteria and fungi into the porous structure of the material. The higher wetting angle hysteresis obtained in the case of BioHPP, indicating its higher roughness, correlates with the roughness of the material measured using a profilometer. The fact that Biocetal is a homopolymer affects its lower roughness in comparison with BioHPP. The arithmetic mean deviation from the mean line (*Ra*) of Biocetal is 0.09 ± 0.01 µm, while for BioHPP it is 0.26 ± 0.19 µm. In the study by Mekkawa et al. [44], the roughness of the material Biodentplast (Bredent, Germany), which is a polymer based on polyoxymethylene (acetal resin), was assessed. The measurement was performed on seven disk-shaped samples with a diameter of 20 mm and a thickness of 3 mm. The samples were made using the injection molding technique according to the manufacturer’s recommendations and then polished using a conventional laboratory polishing technique on one side, while the other side was left unpolished. The average surface roughness (*R_a_*) of the samples before polishing was 1.66 ± 0.45 µm, while after polishing the surface it was 0.18 ± 0.02 µm. The roughness result of the acetal polished using the conventional technique obtained in the study differs slightly from the roughness of Biocetal in our own study (whose roughness is 0.09 ± 0.01 µm). The reason for the difference may be different injection parameters of the Biodentplast product, which may differ from the parameters of Biocetal used in our own study.

In the study by Mekawa et al. [45], the roughness of a removable partial denture made of BioHPP was examined. The calculation of the average roughness was performed by measuring five times each place on the partial denture on its lingual connector: in the middle along the midline, 1 mm to the right and parallel to the midline, and 1 mm to the left and parallel to the midline. The average roughness calculated in this way was 0.86 ± 0.16 µm. This measurement was performed on the polished surface of a removable denture made of BioHPP.

Batak et al. [46] investigated the roughness before and after polishing of milled PEEK-based polymer disks. They compared Coprapeek (100% PEEK) (Whitepeaks Dental Systems, Hamminkeln, Germany), Juvora (100% PEEK) (Juvora, Thornton Cleveleys, UK) and BioHPP (80% PEEK, 20% microceramic filler) (Bredent, Germany). Mory et al. [47] investigated the roughness of CAD/CAM-fabricated implant-supported full-arch fixed BioHPP dentures. In contrast to the veneer surface, the mucosal side of the denture was not coated with the aesthetic composite Crea.lign (Bredent, Germany). The roughness of the mucosal surface was measured using an optical profilometer Alicona Infinite Focus XL200 G5 (Alicona Imaging, Raaba, Austria). The obtained roughness result was 0.66 ± 0.07 µm.

The results obtained by other authors are difficult to compare precisely due to the different types of sample production. In the case of our study, the samples were made by injection molding, while the BioHPP prosthesis frame in the study by Mekawy et al. [45], the BioHPP samples in the study by Batak et al. [46]. and the fixed prosthesis in the study by Mory et al. [47] were obtained by milling the discs using five-axis milling machines. The BioHPP roughness result (*R_a_*) obtained in our study was 0.26 ± 0.19 µm, which is the lowest result among the studies of other authors (Mekawy et al. [45]—0.86 ± 0.16 µm, Batak et al. [46]—0.678 ± 0.225 µm before polishing and 1.186 ± 1.412 µm after polishing, Mory et al. [47]—0.66 ± 0.07 µm).

The total profile height, which is the sum of the height of the highest peak and the depth of the lowest depression within the measurement section (*R_t_*), was 1.07 ± 0.22 µm for Biocetal and 2.76 ± 2.20 µm for BioHPP. Also, the third parameter defining the surface roughness, which is the highest roughness profile height, i.e., the sum of the arithmetic mean of the height of the five highest peaks above the mean line and the average depth of the five lowest depressions below the mean line (*R_z_*), was more favorable for Biocetal (0.78 ± 0.09 µm) in comparison with BioHPP (2.03 ± 1.46 µm). All these results indicate a higher roughness of BioHPP. As in the case of contact angle measurements, the lack of mechanical processing of the tested materials has a significant effect on the surface roughness. Smoothing and polishing the surface of dentures made of these materials reduces their roughness and consequently reduces the risk of colonization by microorganisms present in the oral cavity.

In the studies by Elawadly et al. [48], which consisted in comparing the wetting angle and surface roughness of three types of PEEKs (unfilled PEEK (UFP), filled with ceramics (CFP) and PEEK reinforced with carbon fibers (CFRPs)) divided into four groups—without surface preparation with a sandblaster and after sandblasting with particles of sizes 50 µm, 110 µm, 250 µm. It was shown that the highest roughness (*R_a_*) measured with a profilometer was found in CFRP prepared earlier by sandblasting with aluminum oxide of size 250 µm and it was on average 2.14 ± 0.25 µm.

In our own study, BioHPP filled with 20% ceramic microfiller obtained an *Ra* value of 0.26 ± 0.19 µm. The percentage content and grain size of the PEEK ceramic filler used in the study by Elawadly et al. is not known. Moreover, when comparing these studies with our own study, it should be noted that in the case of the study by Elawadly et al., the samples were milled from blocks using a five-axis milling machine. In our own study, samples were used that were created by injecting melted materials into an injection mold with polished sockets; therefore, due to different methodologies, it is not possible to precisely compare the obtained results. However, it can be observed that sandblasting with aluminum oxide with a particle size of 50 µm is the most effective in smoothing the surface of PEEK with ceramic filler.

In the same study, the authors also measured the dynamic contact angle. The angle measurement technique was different from that used in our study. The advancing angle was read 5 s after applying a 0.1 mL water drop. The receding angle was read after 45 s.

Based on the above results, it can be observed that in both cases (CFP and UFP) the smallest angle hysteresis, and consequently the least rough surface, is observed for samples before sandblasting. PEEK without filler has a significantly larger progressive wetting angle, except for sandblasting with particles of 50 μm diameter.

SEM is a type of advanced microscope in which a beam of electrons accelerated in an electric field with a potential of 1–30 kV and then focused by electromagnetic lens scans the surface of the sample, reflecting from its surface and generating a signal that is processed into an image by the detector. The images generated by SEM are very detailed, which allows for the examination of the surface structure of the sample at the microscopic level. The aim of the study using a scanning electron microscope (SEM) was to comparatively analyze the structure of the tested materials. The observed difference results from the fact that Biocetal is a homopolymer, and BioHPP is a composite. Materials assessed with the naked eye, macroscopically, showed a smoother and more shiny structure of acetal material compared to the matte BioHPP. Microscopically, clusters of ceramic filler are visible in the case of BioHPP and a relatively smooth surface of Biocetal, indicating its greater homogeneity.

From the available literature, it is known that microbial adhesion is a complex phenomenon dependent on factors such as surface roughness, the ability of materials to adsorb water and proteins contained in saliva, which facilitate microbial adhesion. In our own study, the number of microbial colonies observed on the surface of Biocetal was significantly greater than the number of colonies on BioHPP. On average, 30 ± 12 colonies were present on Biocetal, while on BioHPP it was 24 ± 10 colonies.

Studies by Bollen et al. [49] have shown that both supragingival and subgingival increases in surface roughness result in faster colonization and plaque maturation. Therefore, the roughness (*Ra*) of all hard surfaces in the oral cavity should be approximately 0.2 µm or less.

The study by Quirynen et al. [50], which assessed the influence of surface free energy and surface roughness on dental plaque formation, showed that both parameters promote its formation, but the influence of surface roughness was more significant than the influence of surface free energy (SEP). The authors explain that reduced bacterial plaque formation on low-SEP surfaces is caused by the reduced binding force of microorganisms to the low-energy surface and is dependent on the selectivity of bacterial adhesion.

It should be emphasized that both materials have both advantages and disadvantages. In the production of skeletal dentures, as previously mentioned, BioHPP works better mechanically, and if the patient’s clinical and financial conditions allow it, it should be used to make them. In the case of smaller financial possibilities, as well as severely crooked teeth, thermoplastic is recommended. Despite the slightly worse biomechanical effect of Biocetal, the use of a denture made of this biocompatible material is more comfortable for the patient due to its lower stiffness, both during the insertion and removal of the restoration, and it additionally eliminates the need for significant grinding of the teeth in order to eliminate undercuts.

The result of our own microbiological study, however, does not correlate with the theory that the rougher material BioHPP is a substrate more predisposing to the settlement and colonization of bacteria. For this study, as in the previous ones, the samples were not mechanically prepared in any way. More bacterial colonies were observed on the smoother, less rough Biocetal than on BioHPP.

## 5. Conclusions

This paper continues the comprehensive investigation of the physiochemical and mechanical/functional properties of two widely used prosthetic materials—Biocetal and BioHPP—offering a comparative analysis of their characteristics and providing valuable insights for dentists and prosthodontists. Next to extensive mechanical performance studies presented in Part I, the current paper focuses on an in vitro examination of the physiochemical properties, including density, water sorption, contact angle, surface roughness and microbiological analysis. The reported results indicate that BioHH exhibits a slightly higher density, attributed to its highly crystalline, tightly packed molecular structure and the inclusion of ceramic microfillers (20% in content). In contrast, Biocetal is comparatively lighter. The BioHHP microstructure likely contributes to its lower water/fluid absorption if compared to Biocetal, although the contact angle measurements confirm that both materials are hydrophilic, with angles, however, approaching 90°. Furthermore, BioHPP is characterized by greater surface roughness than Biocetal, which is attributed to the presence of ceramic microfiller, but promotes greater abrasive wear of the material in dry conditions. Despite the increased roughness, the composite demonstrates lower wettability, which likely results in reduced adhesion of bacterial colonies and fungi, including *Candida albicans* genus. The overall findings demonstrate that both BioHPP and Biocetal are high-performance materials with notable similarities when their physiochemical properties are considered, but with more pronounced differences in their mechanical behavior. Following this differentiated characterization of both materials, the authors intend to continue their research by conducting mechanical tests simulating the forces occurring during chawing, as well as creep resistance tests.

## Figures and Tables

**Table 1:** SFE values of individual components for measuring liquids for the Owens–Wendt method.

Measuring Liquid	$\gamma_{L}$ [mJ/m^2^]	$\gamma_{L}^{d}\;$[mJ/m^2^]	$\gamma_{L}^{P}\;$[mJ/m^2^]
Distilled water	72.8	21.8	51.0
Diiodomethane	50.8	50.8	0

**Table 2:** Summary of the obtained results of physicochemical tests.

	Medium Density [g/cm^3^]	Boiling Water Absorption [%]	Cold Water Absorption [%]
Biocetal	1.41 ± 0.01	1.27 ± 0.04	0.63 ± 0.05
BioHPP	1.49 ± 0.01	0.41 ± 0.22	0.11 ± 0.02

**Table 3:** Summary of the obtained results of static contact angle and Surface Free Energy (SEP) at 5 s of testing.

	Wet Angle [°]		Surface Free Energy [mJ/m^2^]		
	Distilled Water	Diiodomethane	γ	$\gamma_{s}^{p}$	$\gamma_{s}^{d}$
Biocetal	85.25 ± 2.02	40.31 ± 1.17	40.31 ± 0.34	2.13 ± 0.69	38.18 ± 0.62
BioHPP	84.12 ± 3.25	37.17 ± 0.50	41.96 ± 0.66	2.43 ± 1.15	39.53 ± 1.73

**Table 4:** Summary of dynamic contact angle results.

	Progressive Angle [°]	Receding Angle [°]	Angle Hysteresis [°]
Biocetal	84.7 ± 2.9	51.5 ± 2.6	33.2
BioHPP	90.6 ± 2.5	46.5 ± 3.7	44.1

**Table 5:** Roughness measurement dimensions.

	*R_a_* [µm]	*R_z_* [µm]	*R_t_* [µm]
Biocetal	0.09 ± 0.01	0.78 ± 0.09	1.07 ± 0.22
BioHPP	0.38 ± 0.19	2.03 ± 1.46	2.76 ± 2.20

**Table 6:** Number of colonies grown on Biocetal and BioHPP.

Species of Microorganism	Number of Colonies Grown	
	Biocetal	BioHPP
*Staphylococcus aureus*	36, 39, 36	32, 28, 22
*Enterococcus faecalis*	22, 21, 28	28, 32, 23
*Escherichia coli*	33, 39, 43	23, 28, 26
*Pseudomonas aeruginosa*	44, 45, 31	31, 31, 35
*Candida albicans*	15, 8, 12	6, 9, 5
Control	0, 0, 0	0, 0, 0

## Data Availability

All the raw data are available from the corresponding author on a reasonable request due to privacy.

## Supplementary Materials

The following supporting information can be downloaded at: https://www.mdpi.com/article/10.3390/ma18030519/s1, Figure S1: The injection molded samples used for the investigations; Figure S2: DSA 100 goniometer (Krüss, Germany) (**a**) and its construction diagram (**b**): 1. Microneedle; 2. Material sample; 3. Camera; 4. Light source; Figure S3: 2 samples of acetal and BioHPP coated with a thin layer of sputtered gold to increase conductivity; Table S1: Statistical analysis of the density determination results; Table S2: Statistical analysis of the results of the boiling water absorption test; Table S3: Statistical analysis of the results of the cold water absorption test; Table S4. Results of determining the arithmetic mean deviation from the mean roughness line (*R_a_*); Table S5: Results of determining the highest roughness height according to the 10 highest measured profiles (*R_z_*); Table S6: Results of determining the total height of the roughness profile (*R_t_*); Table S7: Statistical analysis of the results of determining the number of bacterial colonies.
