# Supplementary material for: Comparison of the Performance Parameters of BioHPP® and Biocetal® Used in the Production of Prosthetic Restorations in Dentistry—Part II: Physicochemical and Microbiological Tests: An In Vitro Study
Source: Materials (Basel). 2025 Jan 23;18(3):519. doi: 10.3390/ma18030519 (PMC11818142; doi:10.3390/ma18030519)
Supplement: Supplementary file 1 [file materials-18-00519-s001.zip › materials-3384230-supplementary.pdf]

Supplementary Information

Comparison of the Performance Parameters of BioHPP® and Biocetal® Used in the Production of Prosthetic Restorations in Dentistry. Part II: Physicochemical and Microbiological Tests: An In Vitro Study

Robert Kowalski [1], Wojciech Frąckiewicz [2,*], Magdalena Kwiatkowska [3], Marcin Adamiak [4], Agata Pruss [5] and Ewa Sobolewska [1]

[1]Department of Dental Prosthetics, Faculty of Medicine and Dentistry, Pomeranian Medical University in Szczecin, Av. Powstańców Wlkp. 72, 70-111 Szczecin, Poland;

[2]Ra-Dent Stomatologia Protetyka, Bolesława Krzywoustego Street 19/5, 70-252 Szczecin, Poland;

[3]Faculty of Mechanical Engineering and Mechatronics, West Pomeranian University of Technology in Szczecin, Av. Piastów 19, 70-310 Szczecin, Poland.

[4]Materials Testing Laboratory – RMT L1, Faculty of Mechanical Engineering, Silesian University of Technology, Konarskiego Street 18a, 44-100 Gliwice, Poland;

[5]Department of Laboratory Medicine, Faculty of Medicine and Dentistry, Pomeranian Medical University in Szczecin, Av. Powstańców Wlkp. 72, 70-111 Szczecin, Poland.

*Correspondence: woj.frackiewicz@gmail.com

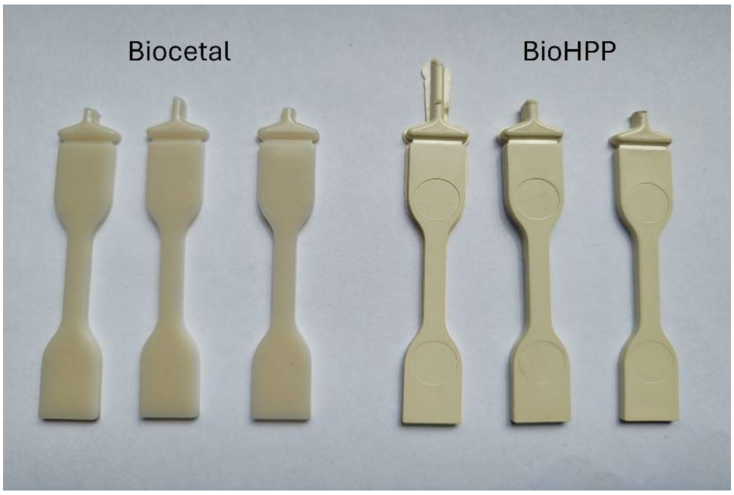

Figure S1. The injection molded samples used for the investigations

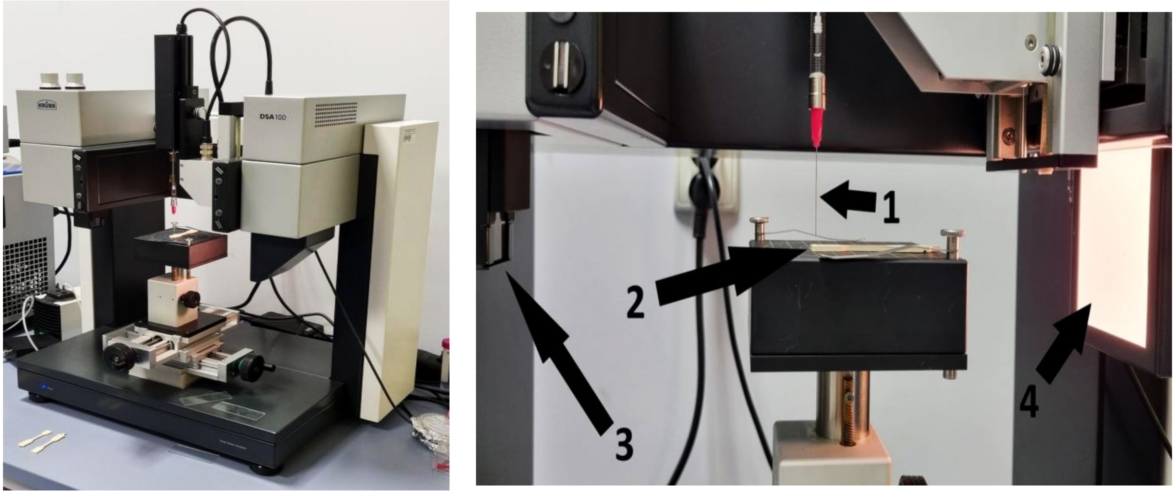

(**a**) (**b**)

Figure S2. DSA 100 goniometer (Krüss, Germany) (**a**) and its construction diagram (**b**): 1. Microneedle; 2. Material sample; 3. Camera; 4. Light source

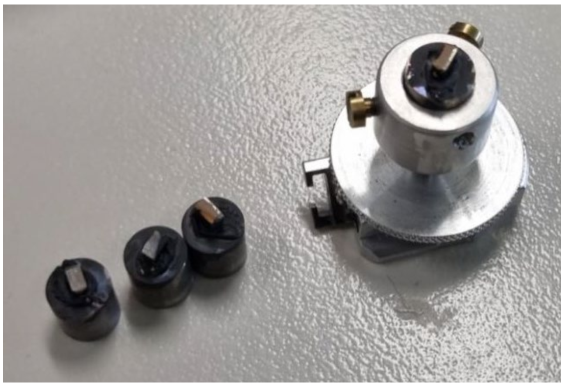

Figure S3. 2 samples of acetal and BioHPP coated with a thin layer of sputtered gold to increase conductivity.

Table S1. Statistical analysis of the density determination results.

Density										
**Material**	n	min	max	mediana	q1	q3	mean	sd	se	statistics
**Biocetal**	5	1,40	1,41	1,41	1,40	1,41	1,41	0,01	0,002	t=-21
**BioHPP**	5	1,48	1,50	1,49	1,49	1,49	1,49	0,01	0,003	p<0,0001

Table S2. Statistical analysis of the results of the boiling water absorption test.

Boiling water absorption										
**Material**	n	min	max	mediana	q1	q3	mean	sd	se	statistics
**Biocetal**	5	1,20	1,32	1,26	1,25	1,31	1,27	0,05	0,02	t=7,62
**BioHPP**	5	0,12	0,63	0,54	0,17	0,60	0,41	0,25	0,11	p=0,002

Table S3. Statistical analysis of the results of the cold water absorption test.

Cold water absorption										
**Material**	n	min	max	mediana	q1	q3	mean	sd	se	statistics
**Biocetal**	5	0,55	0,71	0,63	0,62	0,64	0,63	0,06	0,03	t=19,2
**BioHPP**	5	0,08	0,13	0,12	0,10	0,12	0,11	0,02	0,01	p<0,0001

Table S4. Results of determining the arithmetic mean deviation from the mean roughness line (R_a).

Designation of the arithmetic mean deviation from the mean roughness line										
**Material**	n	min	max	mediana	q1	q3	mean	sd	se	statistics
**Biocetal**	5	0,08	0,11	0,09	0,09	0,09	0,09	0,01	0,01	t=-1,78
**BioHPP**	5	0,11	0,91	0,15	0,12	0,61	0,38	0,19	0,16	p=0,150

Table S5. Results of determining the highest roughness height according to the 10 highest measured profiles (R_z).

Marking the highest roughness height according to the 10 highest measured profiles										
**Material**	n	min	max	mediana	q1	q3	mean	sd	se	statistics
**Biocetal**	5	0,66	0,93	0,77	0,73	0,79	0,78	0,09	0,04	t=-1,72
**BioHPP**	5	0,96	4,88	1,33	1,14	1,85	2,03	1,46	0,73	p=0,159

Table S6. Results of determining the total height of the roughness profile (R_t).

Designation of the total height of the profile										
**Material**	n	min	max	mediana	q1	q3	mean	sd	se	statistics
**Biocetal**	5	0,85	1,48	1,04	0,89	1,07	1,07	0,22	0,11	t=-1,54
**BioHPP**	5	1,26	7,12	1,82	1,50	2,11	2,76	2,20	1,10	p=0,198

Table S7. Statistical analysis of the results of determining the number of bacterial colonies.

Number of bacterial colonies										
**Material**	n	min	max	mediana	q1	q3	mean	sd	se	statistics
**Biocetal**	5	12,00	40,00	37,00	24,00	38,00	30,20	12,00	5,35	t=0,869
**BioHPP**	5	7,00	32,00	27,00	26,00	28,00	24,00	9,77	4,37	p<0,0001
